# Electrospun Oxygen Scavenging Films of Poly(3-hydroxybutyrate) Containing Palladium Nanoparticles for Active Packaging Applications

**DOI:** 10.3390/nano8070469

**Published:** 2018-06-27

**Authors:** Adriane Cherpinski, Melike Gozutok, Hilal Turkoglu Sasmazel, Sergio Torres-Giner, Jose M. Lagaron

**Affiliations:** 1 Novel Materials and Nanotechnology Group, Institute of Agrochemistry and Food Technology (IATA), Spanish Council for Scientific Research (CSIC), Calle Catedrático Agustín Escardino Benlloch 7, 46980 Paterna, Spain; adricherpinski@iata.csic.es (A.C.); melikegozutok@gmail.com (M.G.); storresginer@iata.csic.es (S.T.-G.); 2 Department of Metallurgical and Materials Engineering, Atilim University, Incek, Golbasi, 06830 Ankara, Turkey; hilal.sasmazel@atilim.edu.tr

**Keywords:** polyhydroxyalkanoates, palladium nanoparticles, packaging, electrospinning

## Abstract

This paper reports on the development and characterization of oxygen scavenging films made of poly(3-hydroxybutyrate) (PHB) containing palladium nanoparticles (PdNPs) prepared by electrospinning followed by annealing treatment at 160 °C. The PdNPs were modified with the intention to optimize their dispersion and distribution in PHB by means of two different surfactants permitted for food contact applications, i.e., hexadecyltrimethylammonium bromide (CTAB) and tetraethyl orthosilicate (TEOS). Analysis of the morphology and characterization of the chemical, thermal, mechanical, and water and limonene vapor barrier properties and the oxygen scavenging capacity of the various PHB materials were carried out. From the results, it was seen that a better dispersion and distribution was obtained using CTAB as the dispersing aid. As a result, the PHB/PdNP nanocomposites containing CTAB provided also the best oxygen scavenging performance. These films offer a significant potential as new active coating or interlayer systems for application in the design of novel active food packaging structures.

## 1. Introduction

An important increasing quantity of plastic waste is being generated yearly for which the precise needed time for its biodegradation is certainly unknown. This environmental awareness has driven the development and improvement of new biodegradable polymers, especially for single-use plastic items [1]. In this sense, polyhydroxyalkanoates (PHAs) are well-known biopolymers that can be produced microbially by a variety of microorganisms as an energy storage mechanism. They exhibit similar performance in terms of mechanical, thermal, and barrier properties than petroleum-derived polymers and, thus, they can potentially replace conventional thermoplastics (e.g., polyolefins) in a wide range of applications [2]. In particular, barrier properties are of fundamental importance for food packaging applications. For instance, there are many food products that are very sensitive to oxidation and, to overcome this issue, packages with reduced oxygen permeability are desirable. Additionally, the water resistance is also important, particularly for plastic materials intended for direct contact with high moisture foodstuff as well as materials to be applied in high humidity conditions during storage and/or transport [3].

Poly(3-hydroxybutyrate) (PHB) is currently the most common representative of PHAs and this biopolymer has been proposed for short-term food applications [4]. However, PHB is a brittle polymer, as its enzymatic polymerization leads to the formation of macromolecules with highly ordered stereochemical structures, resulting in a large degree of crystallinity [5]. One of the great advantages of PHB over many other biodegradable polymers is its biodegradability under aerobic as well as anaerobic conditions [6]. For this reason, PHB and their blends with other biopolymers, for instance polylactide (PLA), have been extensively investigated for food packaging applications [7,8,9]. One of the potential application fields of these materials is the development of films for packaging applications. As an example, Zhang et al. [10] studied PHB/PLA blends in different ratios and concluded that by blending PLA with 25 wt. % of PHB some interactions between both biopolymer matrices can be achieved. Furthermore, their results also showed improved mechanical properties.

Palladium nanoparticles (PdNPs) are well known by their ability to dissociate hydrogen molecules to single atoms. This fact is further enhanced due to its nano-sized form and resultant high surface-to-volume ratio [11]. It has been demonstrated that the oxygen scavenging activity of palladium-based oxygen scavenging films is strongly dependent on the coating substrate as well as on the palladium deposition thickness. Optimization of these parameters can result in active scavenging films where the residual headspace oxygen of packaged foods can be scavenged very quickly [10]. There is a drive to find ways to incorporate active packaging technologies directly into the package walls. In spite of the advantages that they offer in maintaining quality and extending shelf life, such systems are still little used. The reason stems from the additional cost involved, the potential toxicity of the added scavenger in the food contact layer, and even more so because of the lack of sufficient technical information on their performance and the lack of understanding of how to apply them effectively [12].

Electrospinning is a fiber production method that employs high electric forces to draw charged threads of polymer solutions or melts up to fiber diameters below 100 nm. It is a low startup cost process in which a wide variety of both polymer and non-polymer materials have been used to form mats composed of nanofibers with a high surface area-to-volume ratio [13]. The electrospinning process has a wide variety of applications such as medical, filtration, tissue engineering, food engineering, packaging, etc. [14,15,16]. Until now, this processing technology remained to a laboratory scale. However, recent developments in instrumentation and process aid design have allowed this process to be scaled to achieve the production volumes required in certain industrial commodity applications such as fortified foods and active packaging [17].

In active packaging, nanotechnology has a significant potential because nanostructures display a high surface-to-volume ratio and specific surface properties. Considering the high surface energy of nanoparticles, which tend to agglomerate and to prevent this aggregation, either polar polymers or surfactants can be used as protective agents and stabilizers of the nanoparticles. This is extremely necessary to obtain mono-dispersed uniform particles and to be, thereafter, used in various application purposes [18,19,20]. The objective of the present study was to prepare and characterize, for the first time, PHB films by the electrospinning process incorporating PdNPs. In order to improve the dispersion of the PdNPs in the PHB matrix, different surfactants were tested.

## 2. Materials and Methods

### 2.1. Materials

Bacterial aliphatic homopolyester PHB was supplied by Biomer (Krailling, Germany) as P226F. According to the manufacturer, this is certified both as compostable and food contact grade, presenting a density of 1.25 g/cm^3^ and a melt flow rate (MFR) of 10 g/10 min at 180 °C and 5 kg. The weight-average molecular weight (M_w_) estimated by the manufacturer was 500 kDa and the polydispersity index (PDI) was 2.

2,2,2-trifluoroethanol (TFE) with 99% purity and _D_-limonene with 98% purity were both purchased from Sigma-Aldrich S.A. (Madrid, Spain). The two tested surfactants, hexadecyltrimethylammonium bromide (CTAB), with 99% purity, and tetraethyl orthosilicate (TEOS), with 98% purity, as well as palladium (Pd) nano-powder, <25 nm particle size measured by transmission electronic microscopy (TEM) and ≥99.5% trace metals basis, were also purchased from Sigma-Aldrich S.A. All products were used as received without further purification.

### 2.2. Electrospinning

A PHB solution for electrospinning was prepared by dissolving the biopolymer at 10 wt. %. The PdNPs were added at 1 wt. % in relation to PHB to the solution. To improve the dispersion of the PdNPs, CTAB and TEOS were also added as dispersing aids at 0.5 wt. % with the PHB and PdNPs mixture. 

Electrospinning was performed using a Fluidnatek^®^ LE50 benchtop line from Bioinicia S.L. (Valencia, Spain) with a variable high-voltage 0–30 kV power supply. This device was equipped with a motorized injector that was scanning towards a metallic collector, aiming to obtain a homogeneous electrospun deposition. The different biopolymers solutions were transferred to a 30-mL plastic syringe and the syringe was connected through polytetrafluoroethylene (PTFE) tubes to a stainless-steel needle (Ø = 0.9 mm) whereas the needle tip was connected to the power supply. The solution was electrospun for 2 h under a steady flow-rate of 6 mL/h using a motorized injector, scanning horizontally towards a metallic grid. The distance between the injector and collector was optimal at 15 cm and the voltage was set at 15 kV. The biopolymer solutions were electrospun in a controlled environmental chamber at 23 °C and 40% relative humidity (RH), for a given processing time and in optimal conditions to achieve steady fiber formation. The processing and solution characterization parameters for the optimal electrospinning of this PHA grade were determined and optimized in a previous work [21].

Finally, the obtained electrospun mats were subjected to an annealing step below the biopolymer’s melting point without applying pressure using a hydraulic press 4122-model from Carver, Inc. (Wabash, IN, USA). The annealing was found optimal at 160 °C, without pressure, for 5 ± 1 s, based on our previous study [21]. The resultant films were air cooled at room temperature. Prior to thermal treatment, the electrospun mats were equilibrated in a desiccator at 25 °C and 0% RH by using silica gel for at least 1 week.

### 2.3. Characterization

#### 2.3.1. Film Thickness

Film thickness was measured with a digital micrometer series S00014, having ±0.001 mm accuracy, from Mitutoyo Corporation (Kawasaki, Japan) at three random positions. The post-processed samples had a thickness of typically 55 ± 4 µm.

#### 2.3.2. Scanning Electron Microscopy

A S-4800 microscope from Hitachi (Tokyo, Japan) was used to observe by scanning electron microscopy (SEM) the morphology of the electrospun PHB fibers and the film cross-sections and surfaces. Cross-sections of the samples were prepared by cryo-fracture of the electrospun PHB films using liquid nitrogen. Then, they were fixed to beveled holders using conductive double-sided adhesive tape, sputtered with a mixture of gold-palladium under vacuum, and observed using an accelerating voltage of 5 kV. Image J Launcher v 1.41 software was used to determine the average fiber diameter and standard deviation by measuring the diameter of at least 100 fibers.

#### 2.3.3. Transmission Electronic Microscopy

The morphology and distribution of the PdNPs was studied using a JEOL 1010 from JEOL USA, Inc. (Peabody, MA, USA) by TEM using an accelerating voltage of 80 kV.

#### 2.3.4. Differential Scanning Calorimetry

Thermal properties of the neat electrospun PHB fibers and films and of the multilayer systems were evaluated by differential scanning calorimetry (DSC) using a Perkin-Elmer DSC 8000 (Waltham, MA, USA) thermal analysis system under nitrogen atmosphere. The measurement was carried out on ~3 mg of each sample using a two-step program from 0 to 200 °C followed by a subsequent cooling down to −50 °C, both at a heating rate of 10 °C/min. The DSC equipment was previously calibrated with indium as a standard and the slope of the thermograms was corrected by subtracting similar scans of an empty pan. Tests were done, at least, in triplicate.

#### 2.3.5. Thermogravimetric Analysis

Thermogravimetric analysis (TGA) was performed in a TG-STDA Mettler-Toledo model TGA/STDA851e/LF/1600 analyzer from Mettler-Toledo, LLC (Columbus, OH, USA). The samples, with an initial weight typically about 15 mg, were heated from 50 to 500 °C at a heating rate of 10 °C/min under nitrogen/air flow.

#### 2.3.6. Infrared Spectroscopy

Fourier transform infrared spectroscopy (FTIR) spectra were collected coupling the attenuated total reflection (ATR) accessory Golden Gate of Specac, Ltd. (Orpington, UK) to Bruker Tensor 37 FTIR equipment (Rheinstetten, Germany). Single spectra were collected in the wavelength range from 4000 to 600 cm^−1^ by averaging 20 scans at a resolution of 4 cm^−1^.

#### 2.3.7. Mechanical Tests

Dumbbell-shaped specimens were die-cut from the electrospun films and conditioned to ambient conditions, i.e., 25 °C and 50% RH, for 24 h prior to tensile testing. Tests were carried out at room temperature in a universal mechanical testing machine AGS-X 500N from Shimadzu Corp. (Kyoto, Japan) in accordance with ASTM D638 (Type IV) standard. This was equipped with a 1-kN load cell and the cross-head speed was set at 10 mm/min. A minimum of six specimens was measured for each sample and the average results with standard deviation were reported.

#### 2.3.8. Water Vapor Permeability

The water vapor permeability (WVP) of the samples was determined, in triplicate, using the gravimetric method ASTM E96-95. To this end, 5 mL of distilled water was placed inside a Payne permeability cup (Ø = 3.5 cm) from Elcometer Sprl (Hermalle-sous-Argenteau, Belgium) to expose the film to 100% RH on one side. The liquid was not in contact with the film. Once the films were secured with silicon rings, they were placed within a desiccator at 0% RH cabinet at 25 °C. The dryness of the cabinet was held constant using dried silica gel. Cups with aluminum films were used as control samples to estimate solvent loss through the sealing. The cups were weighted periodically using an analytical balance with a ±0.0001 g accuracy. Water vapor permeation was calculated from the steady-state permeation slopes obtained from the regression analysis of weight loss data vs. time, and the weight loss was calculated as the total loss minus the loss through the sealing.

#### 2.3.9. d-Limonene Permeability

Permeability to limonene vapor was measured as described above for WVP. For this, 5 mL of d-limonene was placed inside the Payne permeability cups. The cups containing the films were placed at controlled environmental conditions, i.e., 23 °C and 40% RH. Limonene vapor permeation rates were estimated from the steady-state permeation slopes and the weight loss was calculated as the total cell loss minus the loss through the sealing. The samples were measured in triplicate and the limonene permeability (LP) values were calculated taking into account the average film thickness in each case.

#### 2.3.10. Oxygen Scavenging Activity

Round-bottom Schlenk flasks from VidraFoc S.A. (Barcelona, Spain) with a PTFE stopcock and a headspace volume of 50 cm^3^ were used for the oxygen scavenging measurements. The flasks contained a valve for gas flushing in and a O_2_-sensitive sensor spot (PSt3, detection limit 15 ppb, 0–100% oxygen) from PreSens (Regensburg, Germany) was glued onto the inner side of the flasks for measuring oxygen depletion. The electrospun fibers and films were cut (5 × 5 cm^2^) and placed into the flasks. The flask was subsequently flushed for 30 s at 1 bar with a gas mixture containing 1 vol. % oxygen, 4 vol. % hydrogen, and 95 vol. % nitrogen, which was provided by Abelló Linde, S.A. (Barcelona, Spain). The oxygen concentration in the cell was monitored by a non-destructive measurement method using the OXY-4 mini (PreSens) multi-channel fiber optic oxygen meter for simultaneous read-out of up to 4 oxygen sensors, used with sensors based on a 2 mm optical fiber. Oxygen concentrations over time were measured by linking the light-emitting (600–660 nm) optical fibers to the flasks inner sensing spots. The sensor emits a certain amount of luminescence depending on the oxygen concentration in the cell that was calibrated to yield concentration by the equipment. All measurements were carried out at 23 °C and at 50% and 100% RH.

### 2.4. Statistical Analysis

The test data were evaluated through analysis of variance (ANOVA) using STATGRAPHICS Centurion XVI v 16.1.03 from StatPoint Technologies, Inc. (Warrenton, VA, USA). Fisher’s least significant difference (LSD) was used at the 95% confidence level (*p* < 0.05). Mean values and standard deviations were also calculated.

## 3. Results and Discussion

### 3.1. Morphology and Optical Properties

#### 3.1.1. Optical Appearance

**Figure 1:**
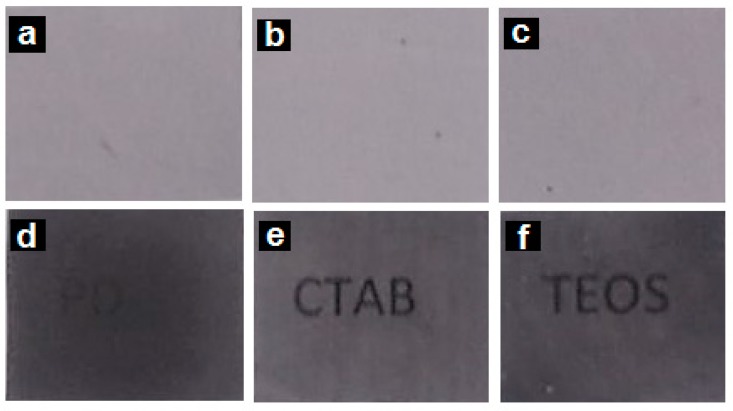
Contact transparency pictures of the electrospun poly(3-hydroxybutyrate) (PHB) fibers containing palladium nanoparticles (PdNPs) and their respective annealed films: (**a**) PHB/PdNP fibers; (**b**) PHB/PdNP/hexadecyltrimethylammonium bromide (CTAB) fibers; (**c**) PHB/PdNP/tetraethyl orthosilicate (TEOS) fibers; (**d**) PHB/PdNP film, (**e**) PHB/PdNP/CTAB film, (**f**) PHB/PdNP/TEOS film.

Figure 1 shows the contact transparency pictures of the electrospun PHB fibers, Figure 1a–c, as well as of their respective annealed films, Figure 1d–f. From these pictures, it can be observed that all the electrospun fiber mats were completely opaque due to the ultrathin size of the fibers that generate a significant level of porosity and hence refract the light very strongly [21]. On the other hand, the annealed films presented an improved contact transparency, specially the sample with CTAB. Due to the presence of the PdNPs, the films developed an expected dark color.

#### 3.1.2. Morphology of Electrospun PHB Materials

**Figure 2:**
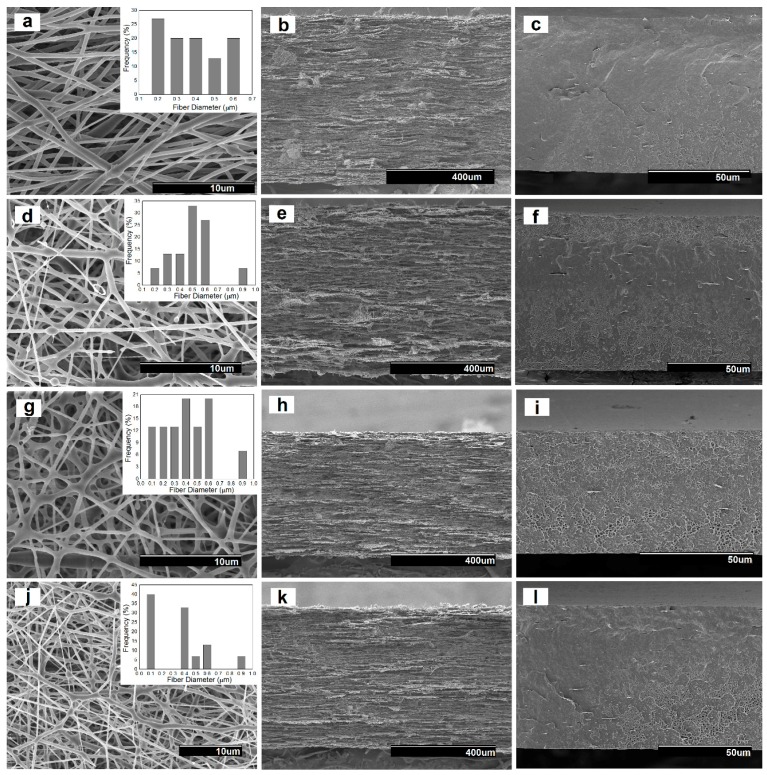
Scanning electron microscopy (SEM) images taken on the surface views and cross-sections of the electrospun poly(3-hydroxybutyrate) (PHB) fibers containing palladium nanoparticles (PdNPs) and their respective annealed films: (**a**) Surface view of the neat PHB fibers; (**b**) Cross-section of the neat PHB fibers; (**c**) Cross-section of the neat PHB film; (**d**) Surface view of the PHB/PdNP fibers; (**e**) Cross-section of the PHB/PdNP fibers; (**f**) Cross-section of the PHB/PdNP film; (**g**) Surface view of the PHB/PdNP/hexadecyltrimethylammonium bromide (CTAB) fibers; (**h**) Cross-section of the PHB/PdNP/CTAB fibers; (**i**) Cross-section of the PHB/PdNP/CTAB film; (**j**) Surface view of the PHB/PdNP/tetraethyl orthosilicate (TEOS) fibers; (**k**) Cross-section of the PHB/PdNP/TEOS fibers; (**l**) Cross-section of the PHB/PdNP/TEOS film.

The morphology of the electrospun fibers and their annealed films were studied by SEM. Representative images of all the electrospun samples are gathered in Figure 2. The images were taken from the surface and cross-sections of the obtained fibers and films. As shown in Figure 2a, the diameter of the neat electrospun PHB fibers was distributed primarily in the range of 200–600 nm, presenting a smooth and bead-free morphology. In particular, the mean diameter was found to be at 350 ± 147 nm. One can observe that the presence of the PdNPs led to a fraction with increased fiber diameter and also resulted in a spindle-type beads formation. This effect can be observed in Figure 2d,g, corresponding to the electrospun PHB/PdNP and CTAB-containing PHB/PdNP fibers, respectively. This observation may suggest that, in some fibrilar regions, partial agglomeration of the PdNPs may occur. Indeed, some degree of agglomeration is a common phenomenon in composites due to the large surface area and high total surface energy associated with nanoparticles incorporated into polymer matrices that makes them amenable to clustering [22]. The nanofibers diameter was previously reported to decrease when the surfactant concentration increased in the electrospinning solution [23]. Interestingly, the TEOS-containing PHB/PdNP fibers, shown in Figure 2j, presented a significant fraction of the fibers with reduced fiber diameter. This effect has been previously described for other electrospun materials and it has been particularly attributed to both the expected decrease in surface tension and an increase in conductivity, which in turn produce an increase in the stretching forces in the jet and consequently decreases the fiber diameter [24,25,26].

From the SEM images of the fibers cross-sections, one can observe that Figure 2b,e, corresponding to the neat PHB and PHB/PdNP fibers, respectively, showed similar morphologies. In particular, both electrospun mats presented cross-sections with relatively high porosity and low compaction. Alternatively, the cross-section of the surfactant-containing PHB/PdNP fibers, included in Figure 2h,k, were seen to be more compacted since the adhesion among the fibers in the layered structure was higher. For all layers, it was also possible to perceive some particles aggregation that may not be necessarily related to the presence of the PdNPs but, more probably, to additives such as the nucleating agent boron nitride, originally included in the biopolymer by the manufacturer.

In the SEM images of the films cross-sections, shown in Figure 2c,f,i,l, it can be observed that the electrospun fibers fused and interconnected among each other after the annealing treatment at 160 °C, successfully leading to the packing of the fiber mat into a continuous film. Among the here-prepared films, the neat PHB film showed a more uniform, smooth, and homogeneous surface. After the addition of the PdNPs and surfactants, the films became more heterogeneous, rougher, and also presented some cavities. This morphology change can be then related to the presence of the PdNPs, which more likely interfered in the fibers coalescence process.

#### 3.1.3. Dispersion of PdNPs

**Figure 3:**
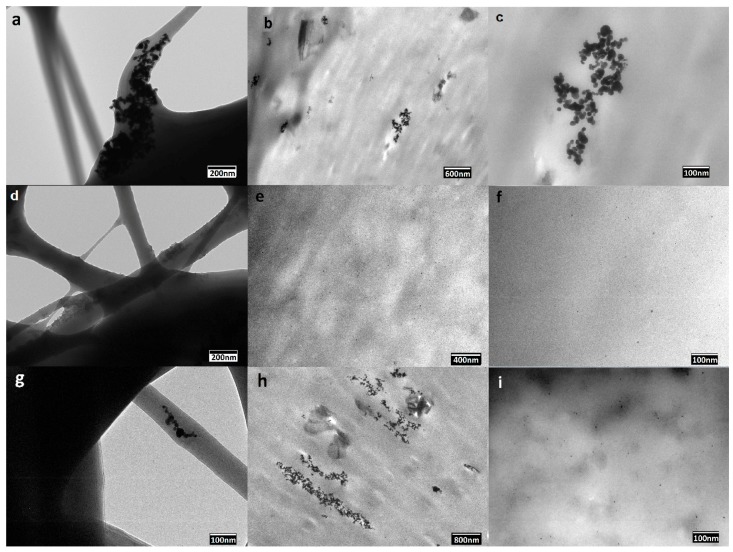
Transmission electron microscopy (TEM) images of the electrospun poly(3-hydroxybutyrate) (PHB) fibers containing palladium nanoparticles (PdNPs) and their respective annealed films: (**a**) PHB/PdNP fibers; (**b**,**c**) PHB/PdNP film; (**d**) PHB/PdNP/ hexadecyltrimethylammonium bromide (CTAB) fibers; (**e**,**f**) PHB/PdNP/CTAB film; (**g**) PHB/PdNP/tetraethyl orthosilicate (TEOS) fibers; (**h**,**i**) PHB/PdNP/TEOS film.

In order to provide a more resolved information about the dispersion of the PdNPs into the PHB biopolymer matrix, TEM was performed on the nanocomposite fibers and films. The distribution of the PdNPs inside the electrospun fibers are illustrated in the TEM images included in Figure 3a,d,g. From the TEM image included in Figure 3a, one can clearly discern that the PdNPs were mainly agglomerated in certain regions of the submicron PHB fibers. One can also observe that the addition of both surfactants, i.e., CTAB and TEOS, as respectively shown in Figure 3d,g, successfully improved the PdNPs dispersion in the PHB fibers. Dispersion and distribution was, however, seen higher in the CTAB-containing sample.

In relation to the annealed films, the PHB/PdNP film without surfactant, shown in Figure 3b,c, still presented a clear aggregation of the particles. As opposite, Figure 3e,f, for the CTAB-containing film sample, and Figure 3h,i, for the TEOS-containing film sample, clearly showed that the PdNPs were evenly and relatively well distributed in the PHB films without forming agglomerates. In the case of the TEOS-containing film sample, the nanoparticle dispersion was less uniform when compared to the sample prepared with CTAB surfactant due to the absence of large grey dark areas. In both PHB films, very small nanoparticles of approximately 5 ± 2 nm can be seen, being homogeneously dispersed along the biopolymer matrix. The present results are in agreement with the results showed by Shaukat et al. [27], where PdNPs were incorporated into polyamide 6 (PA6)/clay nanocomposites and the nanoparticles were largely separated from each other and oriented in all possible directions in the polymer matrix. However, due to the absence of surfactant, some particles still agglomerated into clusters of bigger sizes that increased at the PA6 interfaces with the nanoclays.

### 3.2. Thermal Properties

#### 3.2.1. Melting Profile

The thermal properties of the electrospun PHB fibers and films containing the PdNPs nanoparticles were firstly investigated by DSC analysis. Table 1 gathers the melting temperatures (T_m1_ and T_m2_) and enthalpies (ΔH_m_) obtained from the first heating run. Likewise, the crystallization temperature (T_c_) was also obtained from the cooling run. One can observe that when thermal annealing was applied to the fibers, the melting profile of the PHB materials, both temperature and enthalpy, slightly decreased. In particular, the T_m_ values of the neat PHB-based fibers varied in the 168–170 °C range, melting in a single peak, whereas these values were around 3 °C lower in their counterpart film samples. In addition, the incorporation of the PdNPs into PHB induced a slight decrease in both the melting temperature and enthalpy as well as resulted in the formation of multiple melting peaks. This observation may suggest that the nanoparticles interfered with the crystallization process of the homopolyester, producing more imperfect crystals composed of thinner lamellae that melted over a wider range at lower temperatures and with lower enthalpies [28]. This effect was more intense in the case of the surfactant-containing film samples, which suggests that the PdNPs were better dispersed then highly influencing the packing process of the PHB chains during cooling. In relation to the crystallization from the melt, the PHB film samples incorporating the PdNPs also showed slightly lower values of T_c_ than the fibers, which confirms that the nanoparticles provided an anti-nucleating effect on PHB during the film formation. In any case, given that PHB is known to exhibit cold crystallization during the dynamic DSC runs, it becomes difficult to discuss with certainty potential changes in crystallinity and crystalline morphology [21,26].

#### 3.2.2. Thermal Stability

**Figure 4:**
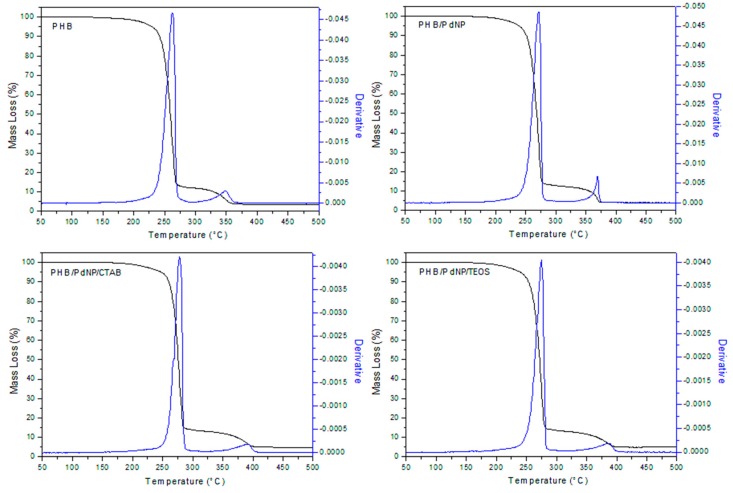
Thermogravimetric analysis (TGA) curves of the electrospun poly(3-hydroxybutyrate) (PHB) and palladium nanoparticles (PdNPs) films with and without hexadecyltrimethylammonium bromide (CTAB) and tetraethyl orthosilicate (TEOS) surfactants.

Figure 4 shows the evolution of the mass loss as a function of temperature for the PHB samples, including the curves of the first derivative analysis (blue lines). As shown in Table 2, it can be seen that the neat PHB initiated degradation at 207 °C while the biopolymer fully decomposed in two steps, seen at 262 °C and 348 °C, providing a residual mass of approximately 3%. Thermal degradation of the PHB films containing the PdNPs and the surfactants also occurred in two stages with a slight increase in thermal stability. In particular, the onset degradation took place in the 220–230 °C range that continued to the first degradation stage at 270–275 °C and, at a slower rate, to over 360–380 °C, remaining a residue of 3–4% of the initial mass of the sample. In this sense, Díez-Pascual et al. [29] demonstrated that the incorporation of zinc oxide (ZnO) improved the heat resistance of PHB, which was ascribed to the barrier effect of the nanoparticles that effectively hindered the diffusion of decomposition products during thermal degradation. Therefore, the PdNPs probably also functioned as a thermal barrier, absorbing heat, thus also resulting in an enhanced thermal stability.

It is also worthy to note the slight increase observed in the thermal stability of the PHB/PdNP films with the incorporation of both surfactants, especially in the case of CTAB. This suggests that the surfactant increased the matrix–nanoparticle interaction, inducing a positive delay in thermal degradation. Taking into account that the ammonium salts degrade at the temperature range of 150–460 °C, the surfactants could successfully provide a bonding effect between both components of the nanocomposite and, eventually, enhance the whole thermal stability of PHAs.

### 3.3. FTIR Analysis

**Figure 5:**
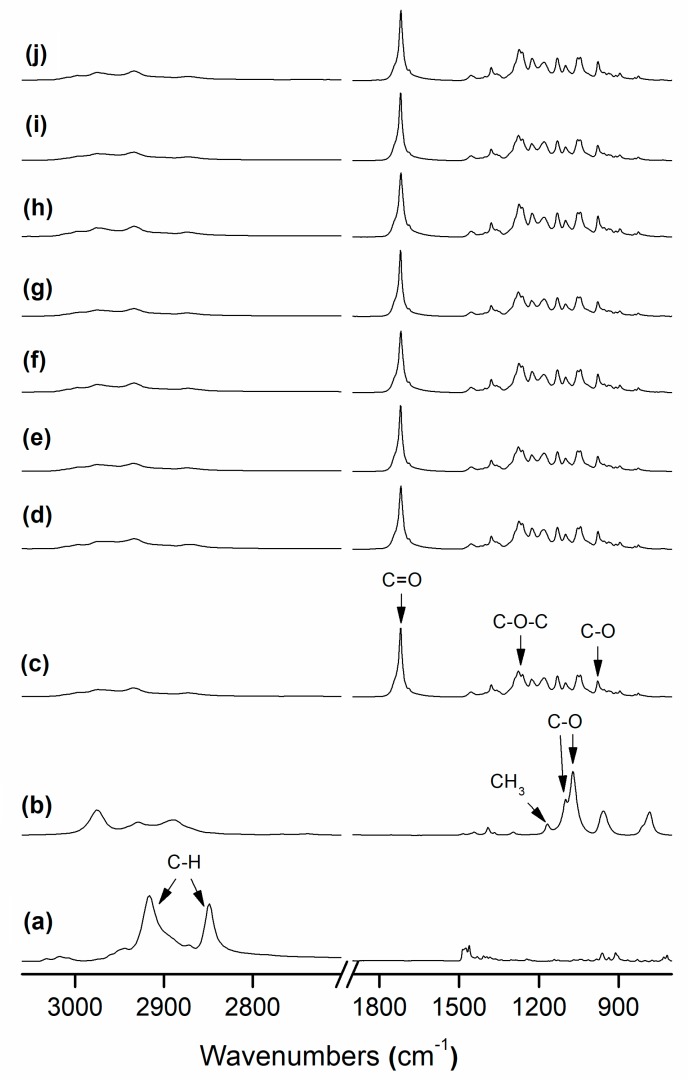
Fourier transform infrared (FTIR) spectra of: (**a**) Hexadecyltrimethylammonium bromide (CTAB); (**b**) Tetraethyl orthosilicate (TEOS); (**c**) Poly(3-hydroxybutyrate) (PHB) fibers; (**d**) PHB film; (**e**) PHB/palladium nanoparticles (PdNP) fibers; (**f**) PHB/PdNP film; (**g**) PHB/PdNP/CTAB fibers; (**h**) PHB/PdNP/CTAB film; (**i**) PHB/PdNP/TEOS fibers; (**j**) PHB/PdNP/TEOS film. Arrows indicate the chemical bonds and/or groups discussed in the text.

FTIR is a powerful tool, which is very sensitive to the molecular environment, to investigate the structural changes that occur in the material during any chemical or physical process. In Figure 5, the FTIR spectra are presented for the above-mentioned PHB-based fibers and films. From the given spectra of the PHB materials, the band centered at 1722 cm^−1^ is an indicative of the C=O stretching vibration for the biopolyester molecule. The absorption bands in the 1200–1300 cm^−1^ range were previously related to the presence of the C–O–C stretching vibrations whereas the band centered at approximately 980 cm^−1^ has been ascribed to stretching bands of the carbon–carbon single bond (C–C) in PHAs [21,30]. In the spectra of the pure surfactants, it is interesting to be noted that the bands at 2873 cm^−1^ and 2977 cm^−1^ correspond to the anti-symmetric C–H stretching of CTAB [19] while the bands located at 1168 cm^−1^, 1103 cm^−1^, and 1080 cm^−1^ have been assigned to the CH_3_ rocking and C–O asymmetric stretching of TEOS, respectively [31]. For the surfactant-containing PHB films, the above surfactant bands or others could not be detected most likely due to the low concentration in which these additives are present in the composite.

Table 3 gathers the band area ratio A1230:A1453 and the 1722 cm^−1^ carbonyl band width at half maximum, which have been recently associated with crystallinity content in electrospun PHB materials [21]. Both a 1722 cm^−1^ band broadening and reduction in the A1230:A1453 band area ratio have been previously connected with a reduction in molecular order and, hence, in crystallinity. Figure 5 and also Table 3 indicate that the band at 1722 cm^−1^ tended to broaden somehow in the case of the film samples as compared to their respective fibers, which can be indicative of a phenomenon of molecular disorder due to thermal treatment in the material. The band ratio seems to be less sensitive, as previously discussed [21], than the carbonyl band. These results may be in close agreement with those previously reported by Pachekoski et al. [32], in which it was indicated that band widening with annealing correlates with the changes in molecular backbone stability and, hence, crystallinity in PHB. Similarly, Mottin et al. [33] also reported a change in crystallinity in PHB by annealing process.

### 3.4. Mechanical Properties

Table 4 presents the mechanical properties, obtained from the tensile tests, of the electrospun PHB films. The incorporation of the PdNPs into PHB caused an increase in both the modulus of elasticity and tensile strength, therefore increasing the elastic deformation and stiffness of the PHB film. The enhancement in mechanical resistance attained in the nanocomposite films can be attributed to the combination of fairly good nanoparticle dispersion and strong interfacial adhesion between both phases through interactions via H-bonding of PHB [29]. In relation to elongation at break, all films presented values of around 3%, which confirms the intrinsic brittleness of PHB. In any case, the presence of the PdNPs did not alter the film ductility and toughness characteristics of PHB while the effect of the surfactants addition on their mechanical performance was also not statistically significant.

### 3.5. Barrier Properties

#### 3.5.1. Water Vapor Permeability

**Figure 6:**
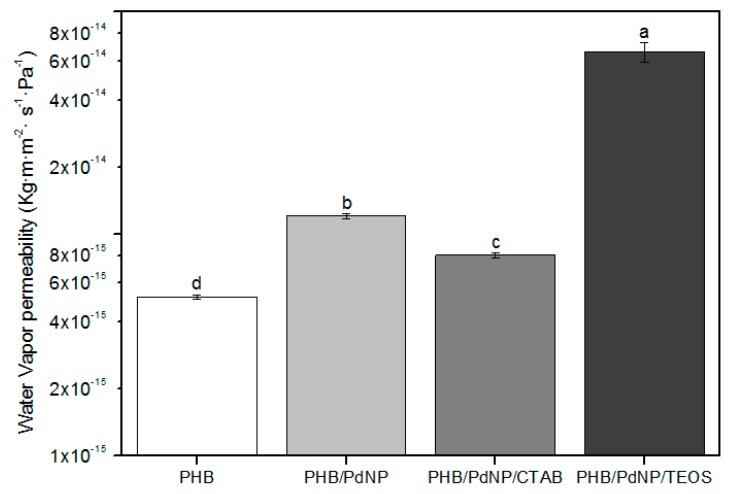
Values of water vapor permeability (WVP) of the electrospun poly(3-hydroxybutyrate) (PHB) and palladium nanoparticles (PdNPs) films with and without hexadecyltrimethylammonium bromide (CTAB) and tetraethyl orthosilicate (TEOS) surfactants. Different letters indicate significant differences among the samples (*p* < 0.05).

Measuring the loss or gain in water content is a common method to estimate the WVP of film samples. The WVP values of the electrospun PHB films are gathered in Figure 6. It can be observed that the neat PHB film showed a higher barrier performance to water vapor than their respective nanocomposites with the PdNPs. In particular, while the neat PHB film showed a WVP value of 5.2 × 10^−15^ kg·m·m^−2^·Pa^−1^·s^−1^, this value was 1.2 × 10^−14^ kg·m·m^−2^·Pa^−1^·s^−1^ for the PHB/PdNP film. The observed permeability increase in the nanocomposite films can be related to the existence of not bonded interfacial regions acting as preferential paths, especially in the vicinity of agglomerates. These preferential pathways could accelerate the diffusion of gas molecules, thus increasing the diffusion coefficient [34]. Interestingly, the WVP values of the PHB/PdNP films was lower in the case of the CTAB-containing film, i.e., 8.0 × 10^−15^ kg·m·m^−2^·Pa^−1^·s^−1^, and considerably higher for the film with TEOS, i.e., 6.6 × 10^−14^ kg·m·m^−2^·Pa^−1^·s^−1^. This result suggests that the dispersion in the PHB/PdNP/CTAB film was higher and, then, the sizes of such unbonded interfacial regions were lower. In addition, the high WPV value observed for the PHB/PdNP/TEOS film suggests that this film sample could be also plasticized by the surfactant, increasing the free volume of the film and favoring the diffusion of water vapor molecules through the film sample [35,36].

#### 3.5.2. d-Limonene Permeability

**Figure 7:**
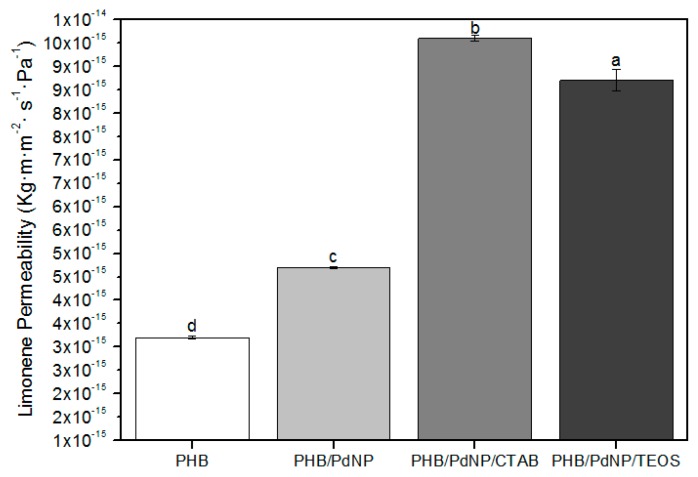
Values of d-limonene permeability (LP) of the electrospun poly(3-hydroxybutyrate) (PHB) and palladium nanoparticles (PdNPs) films with and without hexadecyltrimethylammonium bromide (CTAB) and tetraethyl orthosilicate (TEOS) surfactants. Different letters indicate significant differences among the samples (*p* < 0.05).

To test the barrier performance for volatile compounds such as aromas, _D_-limonene is commonly used. Figure 7 shows the values of LP, where one can observe that the neat PHB film presented the lowest permeability for d-limonene with a value of 3.2 × 10^−15^ kg·m·m^−2^·Pa^−1^·s^−1^. In the case of the nanocomposite films, the LP values increased from 4.7 × 10^−15^ kg·m·m^−2^·Pa^−1^·s^−1^, for the PHB/PdNP film, to 9.6 × 10^−15^ and 9.0 × 10^−15^ kg·m·m^−2^·Pa^−1^·s^−1^, for the CTAB- and TEOS-containing PHB/PdNP films, respectively. As discussed above, the presence of the PdNPs and their agglomerates may result in the creation of preferential paths for sorption and diffusion of the aroma molecules hence resulting in a reduced barrier performance. The here-obtained results are showing opposite behavior as the ones reported earlier by Busolo and co-workers [37] who dispersed silver nanoparticles (nAg) in PLA, yielding nanocomposites with enhanced barrier properties. Similarly, Rhim et al. [38] reported agar/nAg composites where they confirmed a substantial improvement in barrier properties of the composite. In the case of the surfactants-containing PHB/PdNP films, it should be also taken into account that permeability of _D_-limonene in PHB is mainly controlled by a solubilization mechanism due to the capacity of PHAs to uptake large amounts of this organic compound [39]. This supports the fact that plasticized PHB materials present increased values of aroma permeability.

#### 3.5.3. Oxygen Scavenging Activity

**Figure 8:**
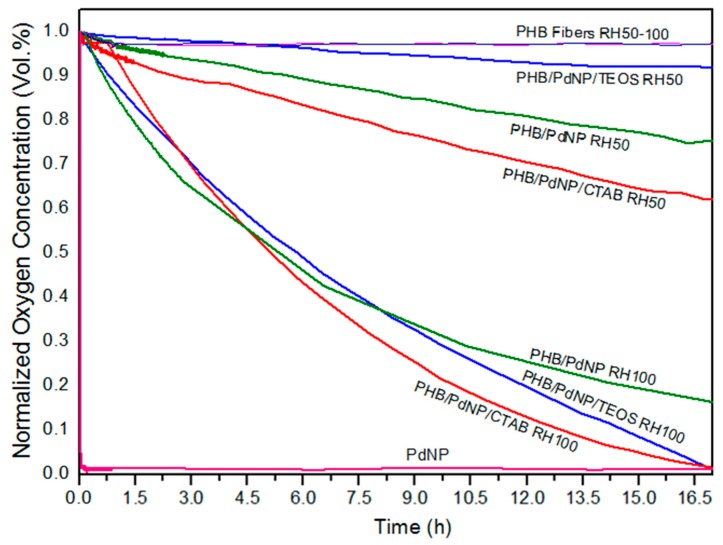
Oxygen depletion of the electrospun poly(3-hydroxybutyrate) (PHB) and palladium nanoparticles (PdNPs) fibers with and without hexadecyltrimethylammonium bromide (CTAB) and tetraethyl orthosilicate (TEOS) surfactants. Values were measured at 50% and 100% relative humidity (RH).

The oxygen scavenging activity of the here-prepared electrospun fibers and films containing the PdNPs was determined by measuring the oxygen scavenging rate (OSR). In relation to the electrospun fibers, Figure 8 shows the decay or depletion of the oxygen concentration as a function of time, for a span time of 800 min, at both 50% and 100% RH. From observation of the graph it can be seen that, while the neat PHB fibers were unable to reduce the amount of oxygen in the cell, comparatively, the free PdNPs in powder form were able to reduce all available headspace oxygen in an extremely short time. The incorporation of the PdNPs into the PHB fibers by electrospinning generated mat samples with intermediate oxygen scavenging activity. However, as it can also be observed in the graph, the performance of the developed nanocomposite fibers was strongly dependent on the RH conditions. All electrospun mats presented a significantly lower oxygen scavenging activity at 50% than at 100% RH. For instance, at the end of the experiment carried out at 50% RH, oxygen depletion varied from almost 40%, for the PHB/PdNP/CTAB fibers, to only ca. 10%, for the PHB/PdNP/TEOS fibers. However, at 100% RH, the electrospun PHB/PdNP mat reached a reduction in the oxygen volume of approximately 85% while both surfactant-containing PHB/PdNP fibers were able to fully consume the whole amount of oxygen after 800 min. It is also worthy to mention that the depletion rate was faster in the case of the PHB/PdNP/CTAB mat, which further confirmed the higher dispersion achieved for the nanoparticles with this surfactant. In relation to the effect of humidity, it is known that moisture favors the catalytic activity of the PdNPs, which can be mainly related to the fact that water can be associatively adsorbed directly on the PdNP surface and thereby interact with the adsorbed hydrogen and oxygen [10]. A possible mechanism is that the adsorbed atomic oxygen and hydrogen forms an OH intermediate that reacts with an adsorbed hydrogen atom or another OH molecule. Another possible explanation is the reaction of adsorbed oxygen with gas-phase hydrogen or with some kind of dihydrogen species weakly adsorbed on the surface. Finally, a concerted reaction of two adsorbed hydrogen atoms and an adsorbed oxygen atom has been also considered [40].

**Figure 9:**
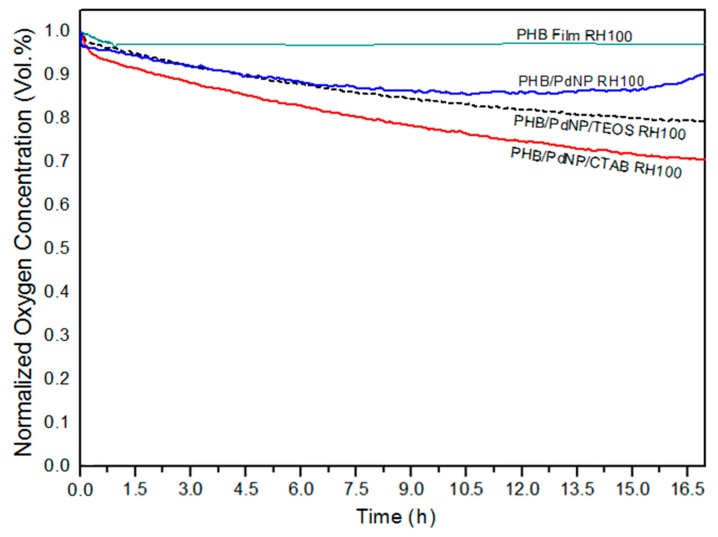
Oxygen depletion of the electrospun poly(3-hydroxybutyrate) (PHB) and palladium nanoparticles (PdNPs) films with and without hexadecyltrimethylammonium bromide (CTAB) and tetraethyl orthosilicate (TEOS) surfactants. Values were measured at 100% relative humidity (RH).

In Figure 9, the oxygen volume depletion obtained with the electrospun films at 100% RH are shown. Comparison between Figure 8 and Figure 9 revealed that the oxygen scavenging effect of the films was considerably lower than that of the same material in the fiber form. This reduction in the OSR is related to the higher surface-to-volume ratio of the electrospun fibers than the films, since the fibers mats present an extremely high porosity. In any case, all PHB/PdNP films still presented significant oxygen scavenging capacity and, among the samples tested, the CTAB-containing films showed the highest performance as expected in view of all the above observations. This result can be explained by the better dispersion of the PdNPs achieved in the PHB matrix using CTAB. In agreement with the data reported here, Ahalawat and co-workers [41] evaluated the simultaneous effects of cationic surfactants on the textural and structural properties of silica nanoparticles. It was observed that the silica nanoparticles displayed better dispersion and lower size than those prepared with other two cationic surfactants using an aqueous TEOS precursor solution with CTAB. In relation to this, it has also been reported that an SiOx matrix between a coating of palladium and the substrate presents higher values of OSR than the palladium coated directly onto PET films [42]. These findings were later confirmed on PLA [10].

## 4. Conclusions

In this study, PdNPs were mixed with CTAB and TEOS surfactants to have better dispersion in electrospun PHB fibers. The resultant fibers were annealed at 160 °C to form continuous PHB films of direct application interest. Morphological analysis carried out by SEM and TEM showed that a better dispersion was achieved for the electrospun PHB/PdNP/CTAB film. DSC indicated that the presence of the PdNPs reduced both the melting point and the degree of crystallinity of PHB, thus acting as an anti-nucleating agent, which was further confirmed by FTIR analysis. WVP and LP measurements indicated that the nanocomposite films, including those modified with surfactants, presented lower barrier performance than the neat PHB film. These results were ascribed to the reduced crystallinity degree and to existence of unbonded interfacial regions and/or voids between the biopolymer matrix and the inorganic nanoparticles that may serve as preferential ways for the diffusion of gas molecules. Finally, the oxygen scavenging activity of the PHB materials was evaluated at different RHs. Although the electrospun films presented lower capacity to absorb oxygen that their counterpart fibers, these still presented significant activity at 100% RH. The here-developed and -characterized electrospun PHB films are suitable potential candidates as coatings or interlayer systems for active food packaging applications and the followed methodology represents a new route to prepare these films due to the relative high dispersion achieved of the PdNPs.

## Figures and Tables

**Table 1:** Thermal properties obtained from the differential scanning calorimetry (DSC) curves in terms of melting temperature (T_m_), normalized melting enthalpy (ΔH_m_), crystallization temperature (T_c_), and normalized crystallization enthalpy (ΔH_c_) for the various poly(3-hydroxybutyrate) (PHB) and palladium nanoparticles (PdNPs) fibers and films with and without hexadecyltrimethylammonium bromide (CTAB) and tetraethyl orthosilicate (TEOS).

Sample	T_m1_ (°C)	T_m2_ (°C)	ΔH_m_ (J/g)	T_c_ (J/g)	ΔH_c_ (J/g)
PHB Fibers	-	169.1 ± 0.9 ^a^	64.1 ± 1.1 ^a^	110.2 ± 0.9 ^a^	59.3 ± 2.0 ^b,c^
PHB Film	-	168.4 ± 1.3 ^a^	71.8 ± 1.3 ^e^	110.5 ± 1.2 ^a^	61.1 ± 0.4 ^a,b^
PHB/PdNP Fibers	156.4 ± 2.1 ^a,b^	168.3 ± 1.1 ^a,b^	59.4 ± 0.9 ^c,d^	108.4 ± 0.5 ^a,b^	63.1 ± 1.5 ^a^
PHB/PdNP Film	155.0 ± 1.2 ^a^	168.4 ± 1.2 ^a,b^	58.5 ± 0.8 ^d^	109.5 ± 2.1 ^a^	56.3 ± 1.0 ^c,d^
PHB/PdNP/CTAB Fibers	156.7 ± 0.5 ^a,b^	167.7 ± 0.9 ^a,b^	62.2 ± 1.4 ^b^	109.1 ± 0.9 ^a,b^	60.3 ± 0.8 ^a,b^
PHB/PdNP/CTAB Film	158.6 ± 1.4 ^c^	165.1 ± 1.4 ^b^	60.3 ± 1.5 ^c^	106.8 ± 0.3 ^b^	55.1 ± 0.7 ^d^
PHB/PdNP/TEOS Fibers	155.2 ± 0.7 ^a^	169.9 ± 0.5 ^a^	59.7 ± 0.9 ^d^	110.8 ± 1.0 ^a^	60.1 ± 1.1 ^a,b^
PHB/PdNP/TEOS Film	159.4 ± 1.9 ^c^	165.5 ± 1.2 ^b^	62.9 ± 0.8 ^a,b^	110.0 ± 2.3 ^a^	51.4 ± 1.8 ^e^

^a–e^: Different superscripts within the same column indicate significant differences among the samples (*p* < 0.05).

**Table 2:** Mean values of thermal stability obtained from the thermogravimetric analysis (TGA) curves of the electrospun poly(3-hydroxybutyrate) (PHB) and palladium nanoparticles (PdNPs) films with and without hexadecyltrimethylammonium bromide (CTAB) and tetraethyl orthosilicate (TEOS) in terms of degradation temperature at 5% of mass loss (T_5%_), degradation temperature (T_deg_), and residual mass at 500 °C (R_500_).

Film Sample	T_5%_ (°C)	T_deg1_ (°C)	T_deg2_ (°C)	R_500_ (%)
PHB	207.0 ± 4.4	262.0 ± 3.8	348.0 ± 6.4	3.08 ± 0.04
PHB/PdNP	224.1 ± 3.6	271.3 ± 2.6	368.2 ± 3.8	3.17 ± 0.03
PHB/PdNP/CTAB	220.0 ± 8.1	278.1 ± 3.4	392.2 ± 5.4	3.81 ± 0.04
PHB/PdNP/TEOS	230.2 ± 2.5	275.2 ± 4.1	386.1 ± 2.3	4.44 ± 0.04

**Table 3:** Full width at half maximum (FWHM) of the carbonyl band centered at ~1722 cm^−1^ and the band area ratio A1230:A1453 for the electrospun poly(3-hydroxybutyrate) (PHB) and palladium nanoparticles (PdNPs) films with and without hexadecyltrimethylammonium bromide (CTAB) and tetraethyl orthosilicate (TEOS).

Sample	FWHM_1722_ (cm^−1^)	A_1230_:A_1453_
PHB Fibers	16.20	4.28
PHB Film	16.35	4.03
PHB/PdNP Fibers	15.10	4.24
PHB/PdNP Film	15.60	4.86
PHB/PdNP/CTAB Fibers	14.44	3.58
PHB/PdNP/CTAB Film	14.23	4.28
PHB/PdNP/TEOS Fibers	15.89	4.25
PHB/PdNP/TEOS Film	16.00	4.28

**Table 4:** Mechanical properties in terms of elastic modulus (E), tensile strength at break (σ_b_), elongation at break (%ε_b_), and toughness (T) of the electrospun poly(3-hydroxybutyrate) (PHB) and palladium nanoparticles (PdNPs) films with and without hexadecyltrimethylammonium bromide (CTAB) and tetraethyl orthosilicate (TEOS).

Sample	E (MPa)	σ_b_ (MPa)	ε_b_ (%)	T (mJ/m^3^)
PHB film *	1104 ± 74 ^a^	17.8 ± 1.8 ^a^	2.9 ± 1.0 ^a^	0.3 ± 0.1 ^a^
PHB/PdNP	1255 ± 15 ^b^	22.5 ± 4.2 ^b^	3.1 ± 1.0 ^a^	0.4 ± 0.1 ^a^
PHB/PdNP/CTAB	1262 ± 14 ^b^	23.3 ± 1.4 ^c^	3.0 ± 0.1 ^a^	0.4 ± 0.1 ^a^
PHB/PdNP/TEOS	1288 ± 230 ^c^	21.7 ± 4.1 ^b^	2.7 ± 1.0 ^a^	0.3 ± 0.2 ^a^

^a–c^: Different superscripts within the same column indicate significant differences among the samples (*p* < 0.05). * Obtained in previous work [21].
